# A Conceptual Framework for 4D Holographic Visualization in Gastrointestinal Endoscopy: Toward Externalized Spatiotemporal Cognition

**DOI:** 10.2196/99446

**Published:** 2026-08-27

**Authors:** Hayato Yamaguchi, Masakatsu Fukuzawa, Takashi Kawai, Fumito Yamanishi, Takahiro Muramatsu, Tadashi Ichimiya, Yasuyuki Kagawa, Kumiko Uchida, Akira Madarame, Takashi Morise, Yoshiya Yamauchi, Shin Kono, Sakiko Naito, Takao Itoi

**Affiliations:** 1 Department of Gastroenterology and Hepatology, Tokyo Medical University, 6-7-1 Nishishinjuku, Shinjuku-ku, Tokyo, 160-0023, Japan, 81 (0)3-3342-6111, 81 (0)3-5381-6654; 2 Hayabusa Internal Medicine Home Clinic, Suginami-ku, Tokyo, Japan; 3 Preventive Medicine Center, Tokyo Medical University, Shinjuku-ku, Tokyo, Japan

**Keywords:** gastrointestinal endoscopy, medical informatics, holography, 4D imaging, human-machine interface, spatial cognition

## Abstract

This viewpoint explores the concept of 4D holographic visualization in gastrointestinal endoscopy and discusses its potential implications from a medical informatics perspective. Current endoscopic practice relies primarily on 2D image displays, requiring operators to mentally reconstruct 3D spatial relationships and temporal changes in a dynamic and deformable anatomical environment. The aim of this viewpoint is to present a conceptual framework that reframes endoscopic visualization as a spatiotemporal information integration problem rather than solely a display problem. We discuss how future visualization systems may integrate spatial and temporal information to support externalized representations of anatomy, endoscope configuration, and procedural dynamics in a shared clinical environment. Potential applications include endoscopic navigation, training, and collaborative procedures. We further discuss key technical and clinical considerations, including information integration, registration stability, real-time responsiveness, workflow compatibility, and uncertainty associated with reconstructed information. This viewpoint highlights spatiotemporal information integration as a central challenge in the development of next-generation visualization systems for gastrointestinal endoscopy.

## Introduction

### Background

Gastrointestinal (GI) endoscopy is essential for the diagnosis and treatment of digestive diseases, with continuous advances in imaging technologies over recent decades [1-3]. Despite these improvements, visualization remains predominantly 2D, requiring clinicians to infer 3D spatial relationships and temporal dynamics from sequential images [4-6].

This limitation is particularly problematic in GI endoscopy due to the inherently dynamic and deformable nature of the GI tract, characterized by peristalsis and respiration and affected by endoscope manipulation. As a result, operators rely heavily on implicit mental reconstruction, increasing cognitive load and contributing to variability in procedural performance, particularly among novice endoscopists [7-9].

These constraints highlight a fundamental mismatch between the spatiotemporal complexity of the target anatomy and the format in which information is presented.

This work reframes endoscopic visualization as an information integration problem rather than a display enhancement problem, emphasizing the need to align visualization paradigms with the spatiotemporal complexity of the target anatomy and its implications for clinical performance and decision-making. The aim of this viewpoint is to present a conceptual framework for 4D holographic visualization in GI endoscopy. Rather than proposing a specific device or implementation pathway, we explore how future visualization systems may integrate and externalize spatial and temporal information to support navigation, training, and collaborative clinical practice. We further discuss the technical and clinical considerations that may influence the development and translation of such systems. This viewpoint is intended for endoscopists, endoscopy trainees, medical informatics researchers, and developers of next-generation visualization systems.

### Why Current Visualization Fails in GI Endoscopy

These limitations are summarized in Table 1, which outlines the key constraints of conventional visualization and their clinical implications. The limitations of conventional visualization in GI endoscopy are fundamentally architectural rather than purely technical. Current systems present information in a format that does not align with the dynamic nature of the target anatomy.

**Table 1.** Limitations of conventional endoscopic visualization and potential contributions of 4D holography.

Limitation of conventional visualization	Clinical implication of conventional visualization	Potential contribution of 4D holography to remove limitation
2D display	High cognitive load	Externalized spatial representation
Lack of depth information	Misinterpretation of lesion depth	Phase-informed or computational depth estimation
Discontinuous temporal understanding	Difficulty tracking deformation	Time-continuous visualization
Implicit scope shape estimation	Loop formation misjudgment	Dynamic spatial representation
Individual mental reconstruction	Variability in skill acquisition	Shared spatial awareness and educational scalability

Key limitations include lack of explicit depth representation, discontinuous temporal perception, dependence on mental reconstruction by the operator, and limited understanding of global scope configuration, such as loop formation [4-6].

Existing approaches—including 3D reconstruction, navigation systems, and mixed-reality (MR) visualization—partially address these limitations. However, most approaches rely on indirect estimation or device-centered visualization and do not fully externalize dynamic spatial information into shared clinical space [5,10-13].

## 4D Holographic Visualization

### Concept of 4D Holographic Visualization

The term “holographic” is used conceptually to describe depth-coherent and phase-consistent spatiotemporal representation rather than a strict physical implementation of holography. We define 4D holographic visualization as a system-level paradigm that integrates depth-resolved spatial structure and continuous temporal dynamics into shared real space.

Digital holography enables the acquisition of both amplitude and phase information, forming the basis of quantitative phase imaging [10-12,14-17]. While primarily applied in microscopic imaging, phase-based approaches provide a conceptual foundation for representing dynamic, deformable biological structures.

**Figure 1.**
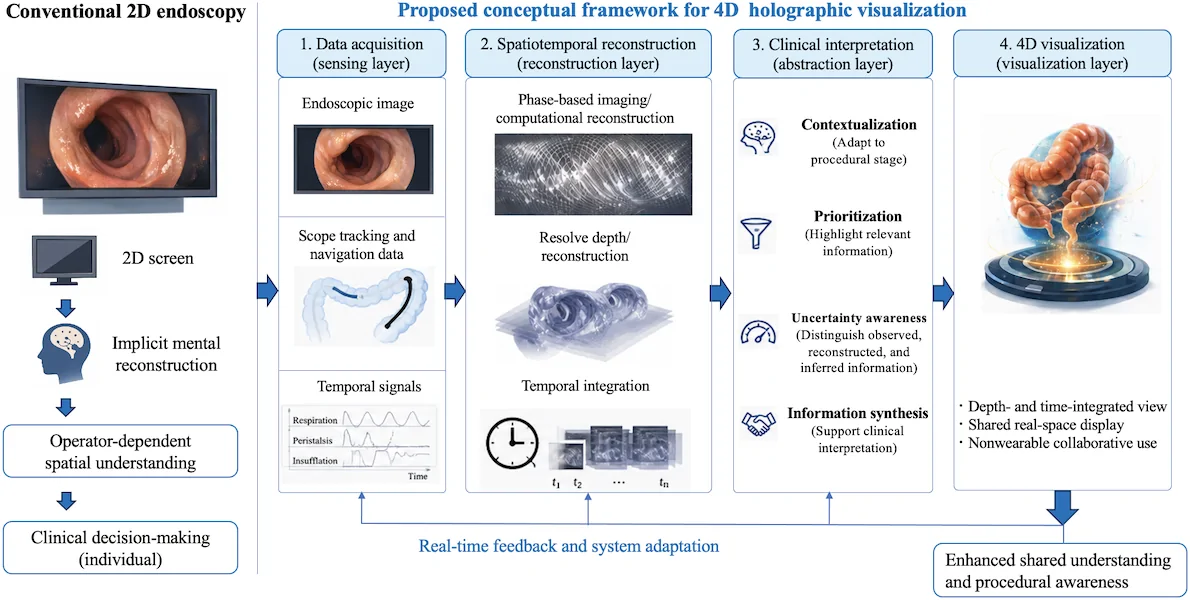
Conceptual shift from mental reconstruction to integrated spatiotemporal visualization in gastrointestinal endoscopy.

This paradigm represents a shift from implicit mental reconstruction to explicit system-supported visualization, effectively externalizing spatial cognition in the clinical environment. This conceptual shift toward externalized spatial cognition is illustrated in Figure 1.

Conventional endoscopy relies on operator-dependent mental reconstruction of spatial and temporal relationships from 2D images. The proposed framework conceptualizes 4D visualization as a 4-layer information integration system consisting of sensing, spatiotemporal reconstruction, clinical interpretation, and visualization layers. The clinical interpretation layer emphasizes contextualization, prioritization, uncertainty awareness, and information synthesis to support meaningful integration of complex spatiotemporal information. This illustration is conceptual and does not represent a specific device or implementation.

### A 4-Layer Information Integration Framework

4D visualization should be understood as an information integration system composed of 4 functional layers: sensing, spatiotemporal reconstruction, clinical interpretation, and visualization. From a medical informatics perspective, these layers define how spatial and temporal information is acquired, reconstructed, interpreted, and externalized for clinical use.

The sensing layer acquires information from multiple sources, including endoscopic images, scope-tracking systems, and temporal signals associated with procedural dynamics. The reconstruction layer transforms these inputs into depth-resolved and temporally coherent representations of anatomy and instrument configuration.

Importantly, reconstructed information alone may not be clinically useful. A clinical interpretation layer is therefore required between reconstruction and visualization. This layer serves to contextualize information according to procedural circumstances, prioritize clinically relevant features, and explicitly distinguish directly measured information from inferred or reconstructed information. Information originating from different sources may carry different levels of reliability and uncertainty, which should be communicated appropriately to users. Such processing may help mitigate information overload and improve the interpretability of complex spatiotemporal data. Rather than displaying all available spatiotemporal information, the purpose of this layer is to transform complex data into clinically actionable representations.

The visualization layer externalizes interpreted information into a shared spatial representation. In this framework, visualization is viewed as the final stage of information integration rather than merely a display modality. The potential clinical value of the system depends not only on the visualization technology itself but also on the effective integration of all preceding layers.

Failures in latency, registration, information interpretation, or workflow compatibility may compromise usability and safety regardless of imaging performance.

**Figure 2.**
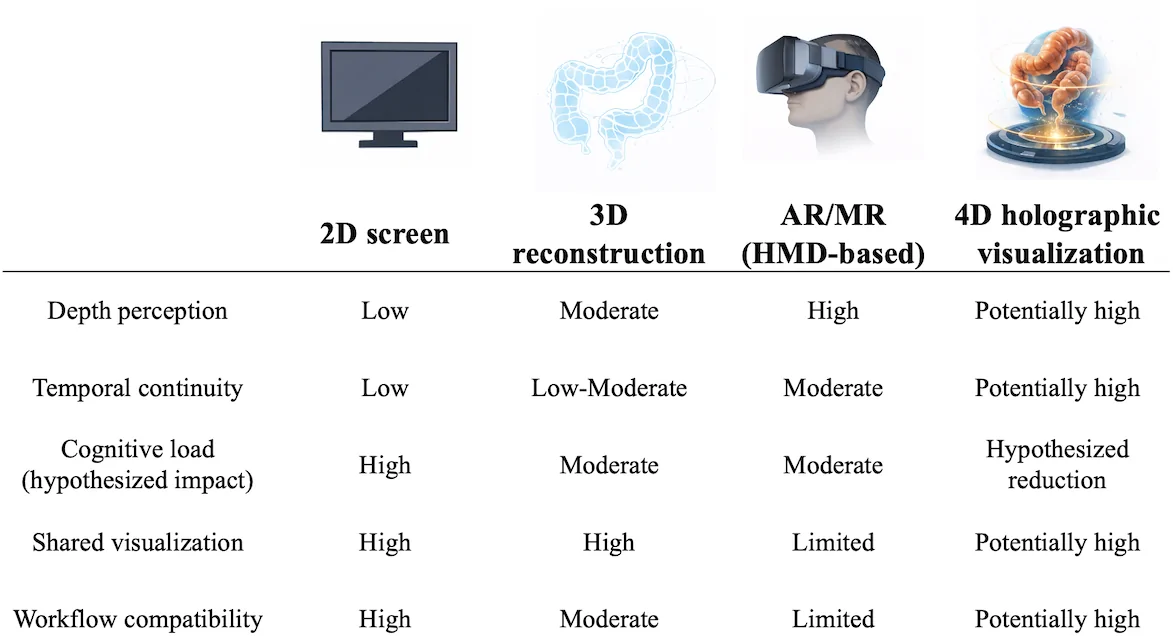
Conceptual comparison of visualization paradigms in gastrointestinal endoscopy. AR: augmented reality; HMD: head-mounted display; MR: mixed reality.

From a system-level and medical informatics perspective, representative visualization paradigms differ not only in display modality but also in how spatial and temporal information is integrated and externalized for clinical use. A conceptual comparison is presented in Figure 2, with corresponding system-level characteristics summarized in Table 2.

Representative paradigms are compared based on hypothesized system-level characteristics. These descriptions reflect conceptual tendencies rather than validated performance metrics and are not intended to indicate superiority. These comparisons are conceptual and intended to illustrate system-level differences rather than quantitative performance.

**Table 2.** Qualitative comparison of system-level characteristics^a^ of visualization paradigms in gastrointestinal endoscopy.

Feature	2D display	3D reconstruction	AR^b^/MR^c^ (HMD^d^)	4D holography
Spatial depth representation	Implicit, mentally inferred	Partial computational reconstruction	Explicit stereoscopic depth	Phase-resolved spatial representation
Temporal deformation handling	Discrete frame–based	Limited continuity	Moderate, latency dependent	Potential for time-continuous integration
Cognitive load	High	Moderate	Moderate	Hypothesized reduction through spatial externalization
Shared visualization	High	High	Limited (device centered)	High (real-space, nonwearable sharing)
Workflow compatibility	High	Moderate	Limited	Potentially high (workflow dependent)
Primary limitation	No explicit depth/time	Incomplete deformation handling	Workflow and sterility constraints	Real-time implementation challenges

a All characteristics represent conceptual hypotheses derived from literature synthesis rather than empirically validated performance metrics.

b AR: augmented reality.

c MR: mixed reality.

d HMD: head-mounted display.

## Clinical Relevance and Use Cases of 4D Visualization

### Loop Recognition and Navigation

Loop formation during colonoscopy represents a major challenge in spatial understanding. 4D visualization may potentially support real-time recognition of scope configuration, improving procedural efficiency and reducing insertion-related difficulty [18-20]. Previous studies of electromagnetic scope imaging have demonstrated improved recognition of scope configuration and enhanced training performance in selected colonoscopy settings, supporting the potential value of externalized spatial information during endoscopic procedures [18-20].

### Support for Novice Endoscopists

By externalizing spatial relationships, 4D visualization may reduce the need for continuous mental reconstruction of anatomy and scope configuration. However, potential benefits are likely to depend on how information is contextualized, prioritized, and integrated into clinically meaningful representations rather than on information quantity alone. Cognitive load has been shown to correlate with procedural performance in endoscopy and simulation-based environments [12,21-24]. However, increasing the availability of spatial and temporal information does not necessarily reduce cognitive burden. Excessive or poorly organized information may instead increase information overload, distract operators, and impair situational awareness. The potential benefits of 4D visualization are likely to depend not only on information availability but also on effective contextualization, prioritization, and interpretation of information. Therefore, the proposed framework does not assume that more information inherently improves performance. Rather, its potential value lies in transforming complex spatiotemporal data into clinically interpretable information through appropriate abstraction and contextualization.

### Shared Spatial Understanding

Unlike head-mounted display–based systems, real-space visualization enables shared perception among multiple operators. This may potentially enhance communication, coordination, and instructional efficiency in team-based procedures [25-28]. Studies from medical informatics and team-based clinical environments have suggested that integrated information displays may improve shared situational awareness and communication efficiency [26].

## Implications for Medical Informatics

This framework positions 4D holographic visualization as a medical informatics problem rather than solely an imaging innovation. It involves integration of spatial and temporal data, optimization of human-machine interaction, and alignment with clinical workflows. This paradigm shifts visualization from displaying images to structuring clinical information.

### Operationalizing the Clinical Interpretation Layer

The proposed framework becomes clinically meaningful only when reconstructed spatiotemporal information is transformed into clinically interpretable representations that support real-time clinical decision-making. While this viewpoint does not prescribe a specific implementation strategy, we propose several design principles to guide the future development of clinically interpretable 4D visualization systems.

Information presentation should be dynamically adapted to the procedural stage rather than remaining static throughout the examination. For example, during colonoscope insertion, a simplified navigation view may display only luminal direction, scope configuration, and loop formation while temporarily suppressing lesion detection overlays or other secondary information. During withdrawal, the visualization would instead prioritize lesion candidates, blind-spot awareness, and inspection completeness while reducing navigational overlays. Such context-dependent adaptation illustrates how the interpretation layer filters, prioritizes, and simplifies reconstructed information according to the operator’s immediate clinical objective rather than displaying all available information simultaneously.

In addition, reconstructed information should remain distinguishable according to its origin. Future systems may integrate directly observed endoscopic images with scope-tracking data, computational reconstruction, preprocedural imaging, and AI-derived predictions. Because these information sources differ in evidential certainty, their provenance and associated uncertainty should be communicated using intuitive visual strategies, such as transparency, confidence indicators, or graphical annotations, enabling clinicians to appropriately interpret reconstructed information.

These design principles position the clinical interpretation layer as the bridge between spatiotemporal reconstruction and clinically actionable visualization, providing an operational and conceptual foundation for future 4D visualization systems.

### Future Directions

**Figure 3.**
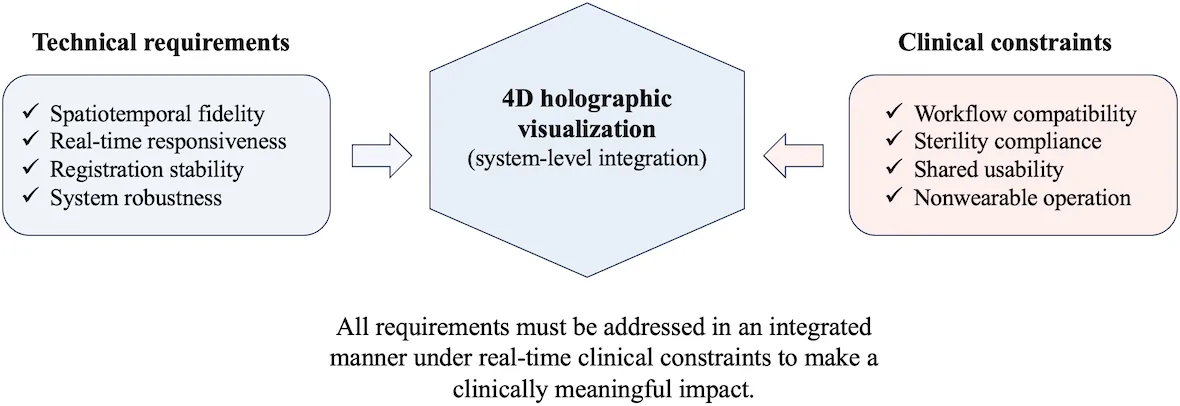
System-level requirements for 4D holographic visualization in gastrointestinal endoscopy.

Several challenges must be addressed before clinical translation, including real-time processing, registration stability, robustness to noise, workflow compatibility, and infection control constraints. Registration represents a particularly important challenge in GI endoscopy because the anatomy is highly dynamic and deformable. Differences in luminal distension, peristalsis, respiration, patient positioning, and endoscope manipulation may result in discrepancies between reconstructed models and the actual anatomical state. Consequently, registration errors may affect the accuracy, interpretability, and clinical utility of integrated spatiotemporal representations. Future systems may therefore require uncertainty-aware registration strategies and mechanisms for communicating registration confidence to users. These challenges may be particularly pronounced when integrating endoscopic data with preprocedural imaging modalities such as computed tomography (CT) or CT colonography, where anatomical conditions may differ substantially from those encountered during real-time endoscopy. Future systems may also require mechanisms for representing source-dependent uncertainty and confidence levels in integrated spatiotemporal models. Future systems may incorporate external imaging modalities or additional sensing technologies, but information derived from these sources should remain explicitly distinguishable from directly observed endoscopic findings and should be presented with appropriate confidence and uncertainty information. Integration with AI-based perception systems may further enhance system performance and usability [29-31]. Beyond visualization alone, future development of 4D information integration systems may facilitate interaction with emerging bioelectronic and intelligent therapeutic platforms. Recent advances in bioinspired electronic skins have demonstrated the integration of multimodal sensing, adaptive interfaces, AI-assisted signal interpretation, and human-machine interaction in a single platform [32]. Such developments suggest potential opportunities for future convergence between spatiotemporal visualization systems and next-generation bioelectronic or therapeutic technologies. Although these applications remain speculative, interdisciplinary integration may represent an important direction for future research. The interdependent technical requirements and clinical constraints necessary for implementation are summarized in Figure 3. These components should be addressed in an integrated manner to achieve clinically meaningful impact. Clinically meaningful impact emerges only when technical requirements and clinical constraints are satisfied simultaneously at the system level.

Future empirical studies are required to validate the proposed framework and quantify its impact on clinical performance and workflow integration. Potential evaluation metrics may include procedural time, error rates, loop formation frequency, cognitive workload (eg, NASA Task Load Index), and training efficiency among novice endoscopists. Importantly, elements of the proposed framework are not entirely novel in isolation. Components such as real-time 3D reconstruction, electromagnetic scope tracking, and MR visualization have already been explored in GI endoscopy. The present framework should therefore be understood as an integration of these existing technological streams into a unified system-level paradigm rather than a completely new standalone technology. From a translational perspective, initial implementation may focus on hybrid systems that combine existing tracking and reconstruction technologies with simplified real-space visualization before achieving fully integrated 4D holographic systems. Such stepwise development may facilitate feasibility testing and clinical adoption. This framework is conceptual and hypothesis generating, and its clinical effectiveness remains to be demonstrated. Potential limitations include system complexity, computational demands, and dependency on stable real-time integration.

## Conclusions

4D holographic visualization represents a system-level reconfiguration of how spatial and temporal information is integrated and presented in GI endoscopy. By externalizing spatiotemporal information into shared real space, this paradigm shifts spatial cognition from the individual clinician to the system level, with implications for procedural performance, training, and collaborative clinical practice. The novelty of this framework lies not in individual technologies but in the explicit system-level integration of spatiotemporal information into shared real-space visualization, which has not been formally conceptualized in GI endoscopy. Although this framework remains conceptual and hypothesis generating, it provides a foundation for future development and evaluation of integrated visualization systems in endoscopy.

## Abbreviations

AR: augmented reality
CT: computed tomography
GI: gastrointestinal
HCI: human-computer interaction
HMI: human-machine interface
MR: mixed reality
